# Critical Periods in the History of the Human Teeth

**Published:** 1901-08-15

**Authors:** C. N. Johnson

**Affiliations:** Chicago, Ills.


### CRITICAL PERIODS IN THE HISTORY OF THE HUMAN TEETH.

BY C. N. JOHNSON, L.D.S., D.D.S., CHICAGO, ILLS.

Abstract of paper read before Tri-State Dental Meeting, Indianapolis, June, 1901.

A study of the history of the teeth of individuals reveals a marked variation in the tendency to decay at different periods of life, and this variation, though not universal nor of constant uniformity, is of sufficient regularity and significance to merit a careful consideration.

We think we know the exciting cause of caries, but of the influences which modify its development we are not so certain. Of the conditions in the mouth which make for or against the tendency to caries we are almost wholly in the dark.

The cause of caries, as demonstrated by Miller, is an acid formed as the result of the propagation of micro-organisms in the mouth; but the reasons why we find the teeth of some individuals extensively attacked by caries, and others practically free from it, have never been sufficiently explained—particularly in view of the fact that we may find micro-organisms in all mouths. Nor do we know why it is that in the same individual there are times when the carious process is exceedingly active, and at others it is almost wholly in abeyance.

Teeth do not grow intermittently hard and soft, nor is there sufficient variation in the structure of the teeth of different individuals to account for the great variation in the tendency to caries. It is then a question of environment more than one of structure, and the conditions surrounding the teeth must be held accountable rather than the teeth themselves.

In view of the case it becomes necessary for us to study carefully the conditions in the mouth, the character of the fluids of the mouth, and to learn, if we may, the particular elements in these fluids which tend to advance or retard the carious process. It is along this line that Michaels seems to have directed his efforts, and in the condensed report of his paper given in the *Dental Cosmos*, of December, 1900, we find suggestive references to the significance of saliva as an index to diathesis.

He says: "As long as we have not discovered the cause of morbid states, and as long as we have not made a more serious study of the essential factors of biochemistry and of the relations of diathesis with the salivary excretion, we will not be in a position to explain in a precise way the morphological differences of dental caries."

When we recognize the fact that the carious process is influenced more by the conditions surrounding the teeth than by the character of the tooth structure itself, we are placed in a more intelligent relation to the matter, and are in a better position to study the factors which bring about the variations which we see.

It is clearly demonstrated that the attempt to produce mere mechanical cleanliness of the mouth is not sufficient.

It would seem that if we are to accomplish anything we must so change the conditions of the mouth that the micro-organism of dental decay can not exist therein. There are agencies at work affecting the life forces of the human economy, the nature of which we know comparatively little. There are individuals in whose mouths caries is seldom seen, while there are others with teeth as well developed, and where even greater care is taken, who lose their teeth bit by bit, despite the most persistent effort to save them. In one case there is a subtle condition present in the mouth which militates against the activity of the micro-organism, while in the other the conditions are favorable to its development. We are wholly unable to distinguish between these two conditions—we can see only the results.

The period of youth is the critical period, and while the age limit can not be set with accuracy, it may be said that in susceptible patients the teeth should have constant supervision by the dentist from the time the deciduous teeth show indications of caries. The desirability of keeping the deciduous teeth as free as may be from decay has frequently received emphasis from writers on dental subjects, and does not require extended repetition here, except to mention two cardinal points which have a direct bearing on the early establishment of immunity.

To keep the teeth and the surrounding tissues in a normal condition, it is necessary to maintain the full functional activity of the parts, and the teeth should be kept sufficiently comfortable to admit of adequate functional use. The part that mastication plays in the prevention of caries, aside from the general health of the patient through digestive influences, and the stimulation of the surrounding tissues through healthy exercise, relates to the mechanical cleansing of the surfaces of the teeth by the friction of food in passing over them. If all of the surfaces were so situated as to be wiped clean by mastication, and if mastication were performed to the full limit, we should seldom see decay of the teeth.

The other point connected with the care of the deciduous teeth relates to the effect which neglected caries has upon the prolongation of susceptibility. It seems to be a fact that in a mouth where it would be the natural order of things for immunity to take place at or near a given age, it is found that the period of immunity is delayed, and the period of susceptibility prolonged by the presence of neglected cavities.

It is not always possible to control caries in the deciduous teeth, but we should fearlessly approach this question of establishing in the mouth of our patients an early immunity from the ravages of decay. Suppress caries as if it were a deadly and contagious disease, of which nature it in fact seems to manifest certain symptoms when it attacks the teeth of children.

In line with such treatment preventive measures should be taken in guarding the permanent teeth while they are erupting in those mouths where a pronounced tendency to decay is manifest. The first permanent molar should in all such cases be the object of especial care, on account of its early eruption and its consequent liability to decay, and also because it is of all others the most important tooth in the mouth. The moment such a tooth shows through the gum, the occlusal surface should be protected from decay by carefully cleansing it with alcohol, and drying out the sulci and fissures, and forcing into them some oxyphosphate of zinc. This will tide the tooth over a very critical period in those cases where the tendency to caries is great.

The most critical period so far as decay of the teeth is concerned may be said to be up to the twentieth year, though many cases exhibit progressive caries past this age, and very many others take on a condition of practical immunity earlier than this.

Illness may change a mouth from an immune to a susceptible one, and we sometimes find a variation in the tendency to decay without being able in any way to account for it. The influence of climate or the change from one country to another. Foreigners coming from Europe to reside in America often manifest an increased tendency to caries, and we have it on the very best of authority that the factory girls in some of our eastern cities coming recently from foreign countries, particularly those from Norway and Sweden, show the most astonishing development in this connection. It is recorded that girls who come here with teeth practically free from caries are frequently known to be attacked so vigorously by this disease as to lose all their teeth in a few years.

In some instances there develops in the mouths of elderly people a marked tendency to decay in teeth which have previously been comparatively free from it. The character of this decay is usually different from that which attacks teeth earlier in life, and is the result of a recession of the gums, whereby the neck and root of the tooth is exposed in such a way that the tooth is girdled with decay at a point root-wise of the enamel. This form of decay is very disastrous and more difficult to control than the ordinary forms where the penetration is deeper at a given point.

The practical lesson to be learned from a study of this entire question of periodical immunity and susceptibility lies in the hope it gives us of eventually combating the disease of dental caries, even in some of the most desperate cases. Patients who are lukewarm on the subject or who are easily discouraged when the carious process seems persistent, it is our duty to so inform them in regard to the history of dental caries as it relates to the expectancy of an approaching immunity, that they may thereby take heart and co-operate with the dentist, to the end that the greatest number of the natural teeth may be saved. The fact is, that it is the rarest exception possible where it ever becomes necessary for an individual to lose a tooth by decay if dentist and patient do their duty. And if this be true, have we not as a profession much to answer for? If we are losing teeth by caries to-day it is either because we are incompetent ourselves, or else because we have not educated our patients up to the point where they will co-operate with us to their own ultimate good.

### DISCUSSION.

Dr. H. A. Smith, of Cincinnati: The period of active encroachment of caries on the teeth we may say extends from the age of three to twenty years.

There is no time during this period when the mouth is free of micro-organisms. The newly-born babe is probably free from micro-organisms, but the minute it opens its mouth or gasps for breath the micro-organisms enter, never to leave. It is fortunate, however, that an absolutely sterile condition is not imperative; carelessness or thoughtlessness in maintaining cleanliness of the mouth will account for most of the caries which destroy the teeth. Children and youths are not as careful in making the mouth toilet as they should be. There is, however, little doubt but incidental and accidental conditions have their influence in propagating dental caries. The diseases of childhood and youth, especially those which affect or modify the glandular secretions, may be prolific causes of dental caries, simply producing conditions in which decay-producing micro-organisms develop most rapidly.

The adult is in the enjoyment of the full vigor of life and throws off disease and withstands disease-producing agencies; his teeth do not therefore decay. Adults also have formed habits of cleanliness and more faithfully carry out toilet requirements, hence their teeth do not suffer, neither do other organs of the body.

Again, teeth of the aged take on decay for reasons that may be as easily traced. Old people, because of body weakness or infirmity, relax in their efforts to keep up accustomed hygienic practices. Because of diseased gums the teeth change position and some are lost and opportunity is made for the lodgment of food, which is allowed to ferment and bring about the natural result of decay of the exposed surfaces of denuded dentin or cementum. It is much more difficult to keep such teeth clean from soft or mucus deposits, to say nothing of the deleterious calcic deposits which result from altered glandular secretions. Even the saliva itself is less powerful in sterilizing qualities.

In an orphan asylum which comes under my notice the children are compelled to systematically brush the teeth and keep their mouths clean, and it is surprising that there is so little dental caries in those children's mouths.

Dr. H. B. Tileston, of Louisville, Ky.: The idea of dental prophylaxis as drawn out by the essayist is the most important theme that we can consider. It is possible that dental caries may be an infectious disease, spreading from one cavity or tooth to another, or contagious, spreading from person to person by contact in various ways. It is not likely that it can be hereditary, except as the tooth tissues are developed of such character that they are predisposed. But the teeth after development are subject to unfortunate vicissitudes, of contact with uncleanly things and persons, the drinking cup, being kissed by uncleanly persons, and in many such ways inocculation may occur. It seems that it is a hopeless task to even think of trying to stamp out a disease which has so many resources that can not be reached by any kind of professional care or advice. There are not enough dentists and never will be to keep peoples' mouths clean. We may do some good by talking and educating our younger patients in ways of cleanliness, but it will take many years to overtake and exterminate the well-intrenched micro-organism. It seems more probable that by the evolution theory and change in our methods of living that the teeth may in time become extinct.

Dr. C. M. Wright, of Cincinnati: The essay is elegantly written and directly to the point. The treatment of the subject suggests to my mind the humoral pathology of Paul Gibbier, who classified the juices of the body as acid, alkaline or neutral. The acid and alkaline juices were associated with diseased conditions, and it was only when the juices were neutral that perfect health was indicated. We should give more attention not only to the local conditions of the mouth but to the salivary secretions as well, not that we may change them so much as that they give us an intelligent conception of the causes of conditions which meet us. It is not an impossible conception that some day we may treat and even control such local diseases as dental caries by systemic therapeutics. The surgical and local treatment should be in this way materially reinforced. We have given much time to the study of the etiology of dental caries and should thoroughly comprehend it in order that we may the more perfectly apply therapeutic remedies.

Dr. G. W. Cook, of Chicago: The essayist has gone to the vital point as a starter in recognizing and treating dental caries as a disease. The saliva is only of interest to us when it exhibits abnormal conditions. In its normal condition as secreted from its glands it contains metallic salts in solution, which are changed or precipitated by the conditions met in the mouth. And these physical and chemical reactions have much to do with producing media favorable or unfavorable to the development or growth of micro-organisms. Changes in normal function are frequent in youth and old age for reasons that are obvious, and may occur accidentally from sickness at any period of life. These ever-changing conditions have much to do with making favorable environment in the mouth for the propagation of disease, and more understanding of them ought to be had so that the teeth may be shielded from their pernicious influences. In childhood these functional disorders interfere with developmental processes to the extent sometimes of producing malformed tissue which readily gives way under the activity of destructive agents which might not seriously damage teeth of more perfect structure.

Dr. D. M. Gallie, of Chicago: The most interesting point in the essay to me is the reference to the critical periods in children's teeth. What can we do to influence parents to look after the teeth of children at a sufficiently early date to insure their integrity until the permanent teeth are ready to take their places? Many times the deciduous teeth are prematurely lost or seriously diseased before the dentist is consulted. Then it is difficult to control the patient or to make any kind of a conservative operation. We are blamed for our incapacity, when the fault is entirely with some one else. Then there comes a time when the mother relaxes in her watch over the boy or girl as she has others to attend to and the boys or girls begin to neglect their teeth, and before any one is aware of it some valuable tooth has gone beyond repair.

I have known of several cases also in which the teeth have suffered by climatic changes. A couple of robust Irish girls put their teeth in my care shortly after arriving in this country, but in spite of my best efforts several teeth were lost before they became acclimated.

The embryonic period is also one of great importance; much may be done in the study of this phase of the subject.

Dr. J. L. Young, of Detroit: It seems, according to our modern scientists, that there are no such things as hard and soft teeth. But our patients think there is a great difference in their teeth at different periods. I am certain every practitioner knows that some teeth yield to instruments much more readily than others, and there will be a variation in the same mouth. There may not be any appreciable difference in their chemical constitution, but physically they undoubtedly vary.

Climate and diet undoubtedly have much to do with the resistant qualities of the teeth. I practiced several years in Montana and had as patients many Swedes who had recently come to this country. It was practically impossible to save their teeth. I used to attribute it not so much to the climate as to the fact that they had changed from a diet of hard, dry food to one of mush and pie. The influence of diet on the systemic nutritive functions may have something to do with changing the character of the salivary juices or even of the nutritive juices of the teeth, but I am inclined to think that the chewing of the hard and dry resistant foods had much more to do with it in keeping the teeth clean and giving them adequate exercise. How often you see people with little or no decay of the teeth who have never used a tooth-brush at all!

Dr. F. W. Sage, of Cincinnati: Dental caries is more likely to occur at some periods than others, in spite of all efforts at maintaining cleanliness of the mouth. It has been said that if we could have perfect cleanliness of the mouth there would be no decay. The reference of the essayist to the work of Dr. Michaels and the discussion here indicate that something more than ordinary cleanliness must prevail if any results are to be expected. Our hopes are again raised to expect some time in the near future when we shall know all about the saliva and how to control it at will; that there shall be positive remedies against micro-organisms and all other causes of caries of the teeth.

Dr. C. N. Johnson: Ordinary local cleanliness during the critical periods is, of course, of some value, but we all know it is insufficient. Systemic disease produce local conditions which ordinary cleansing will not combat. The idea that caries can be transmitted by kissing, for instance, is probably erroneous. The germs must find a suitable environment in which to grow. The chemical character of the saliva has much to do with it. Germs will not proliferate in an excessively acid or alkaline saliva. There must be provided a suitable place for the lodgment and activity of the micro-organisms. If any of these factors are wanting, in just so far the conditions are unfavorable. Patients are at fault in not consulting the dentist more often, but dentists are too often to be blamed for not putting the teeth in such condition as will make the environment most unfavorable for the development of micro-organisms. A dentist can do much in restoring decayed teeth to normal contours with well-adapted filling materials, polishing and shaping the filling of teeth so that they will not serve as places for the accumulation of food stuffs or other detrimental substances. Dentists should have the highest ambition to place their patients' teeth in such condition that they may be most easily and readily kept clean by the normal functions of the mouth.

